# Decoding the temporal dimension of innate immunity in intravesical BCG therapy

**DOI:** 10.3389/fimmu.2026.1904049

**Published:** 2026-07-22

**Authors:** Xuewen Diao, Bangwei Che, Jiancheng Zhai, Bo Shao, Kaifa Tang

**Affiliations:** 1 The First Clinical Medical College, Guizhou University of Traditional Chinese Medicine, Guiyang, China; 2 Department of Urology and Andrology, The First Affiliated Hospital of Guizhou University of Traditional Chinese Medicine, Guiyang, China

**Keywords:** BCG, bladder cancer, immunotherapy, innate immunity, kinetic profile

## Abstract

Innate immune signals encode functional instructions through temporal patterns such as oscillation frequency and duration—a principle validated at the single-cell level. Yet clinical evaluation of host responses in tumor immunotherapy remains dominated by “how strongly” the immune system is activated, while the waveform of the response—how it unfolds and when it resolves—is largely overlooked. This disconnect raises unresolved questions: why can tumor progression persist despite intense inflammation, and why is fixed-interval maintenance not universally effective? Intravesical BCG instillation offers a way forward: each instillation is a controlled stimulus, voided urine provides a noninvasive sampling window, and oncological plus bladder-function outcomes form a “controlled stimulus–noninvasive sampling–dual-endpoint” closed loop. Using this model, we propose a hypothesis organized around two observation levels. The fundamental unit is the single−instillation single−cytokine waveform—the complete concentration-time curve of one cytokine following one BCG dose; the second is the longitudinal waveform trajectory, which captures how such waveforms evolve across repeated instillations. Both levels reside at a mesoscopic interface between single-cell signaling dynamics and macro-scale clinical outcomes. Our central hypothesis is that temporal features of a single-instillation single-cytokine waveform—time-to-peak, elimination half-life, and decay morphology—carry predictive information independent of peak amplitude. Distinct waveform shapes may differentially instruct adaptive immune quality, while the decay phase defines the time window for immune homeostasis restoration, thus providing a biological rationale for individualized instillation timing. If validated, this framework would expand the evaluation paradigm for intravesical immunotherapy from an “amplitude” dimension to a dual “amplitude + waveform” paradigm.

## 1 Introduction

The innate immune system does not respond to stimuli in a simple “on/off” fashion; it encodes distinct functional instructions through dynamic patterns such as oscillation frequency, duration, and decay kinetics (1, 2). How an immune response unfolds and when it resolves is itself a critical information carrier that determines response outcome (3). Yet in clinical tumor immunotherapy, the evaluation of host responses remains dominated by a single question: how strongly is the immune system activated? The kinetic dimension of the endogenous host response—its temporal shape—has received little systematic attention.

This neglect is not purely conceptual; it reflects a methodological bottleneck. For most solid tumors, continuous, high-frequency, noninvasive sampling of the local immune microenvironment is hardly feasible. Intravesical Bacillus Calmette-*Guérin* (BCG) therapy for non-muscle-invasive bladder cancer offers a way through this impasse. Each instillation is a controlled immune stimulus; voided urine provides a noninvasive, repeatable sampling window; and decades of clinical experience have generated dual-endpoint data encompassing both oncological outcomes and bladder function. This “controlled stimulus–noninvasive sampling–dual-endpoint” closed loop makes the bladder a unique model for observing innate immune response kinetics directly in the human body.

In this article, we propose a hypothesis centered on two observational levels. The first, and the fundamental unit of analysis, is the single-instillation single-cytokine waveform (see **Box 1** for key terms): the complete concentration-versus-time curve of one specific cytokine (e.g., IL-6, IL-8) in urine following a single BCG instillation—from baseline rise through peak to decay. The second is the longitudinal waveform trajectory: the evolution of single-instillation waveform features across repeated instillations over the course of induction and maintenance therapy, reflecting whether the immune system undergoes sensitization, tolerance, or exhaustion.

Box 1 **Glossary of key terms**

**Table:** 

Terms	Definition
Temporal coding	The encoding of functional instructions by immune signals through kinetic patterns—such as oscillation frequency, duration, and decay rate.
Single-instillation single-cytokine waveform	The complete concentration-versus-time curve of one specific cytokine in urine following a single BCG instillation, from baseline through peak to decay.
Longitudinal waveform trajectory	The evolution of single-instillation waveform features across repeated instillations over a full treatment course.
Mesoscopic profile	The population-level, noninvasively observable cytokine concentration-time curve in urine, intermediate between single-cell signaling dynamics and clinical outcomes.
Waveform parameters	Quantifiable metrics extracted from a single-instillation single-cytokine waveform: Cmax, Tmax, T1/2, AUC, and decay morphology classification.

**Figure 1:**
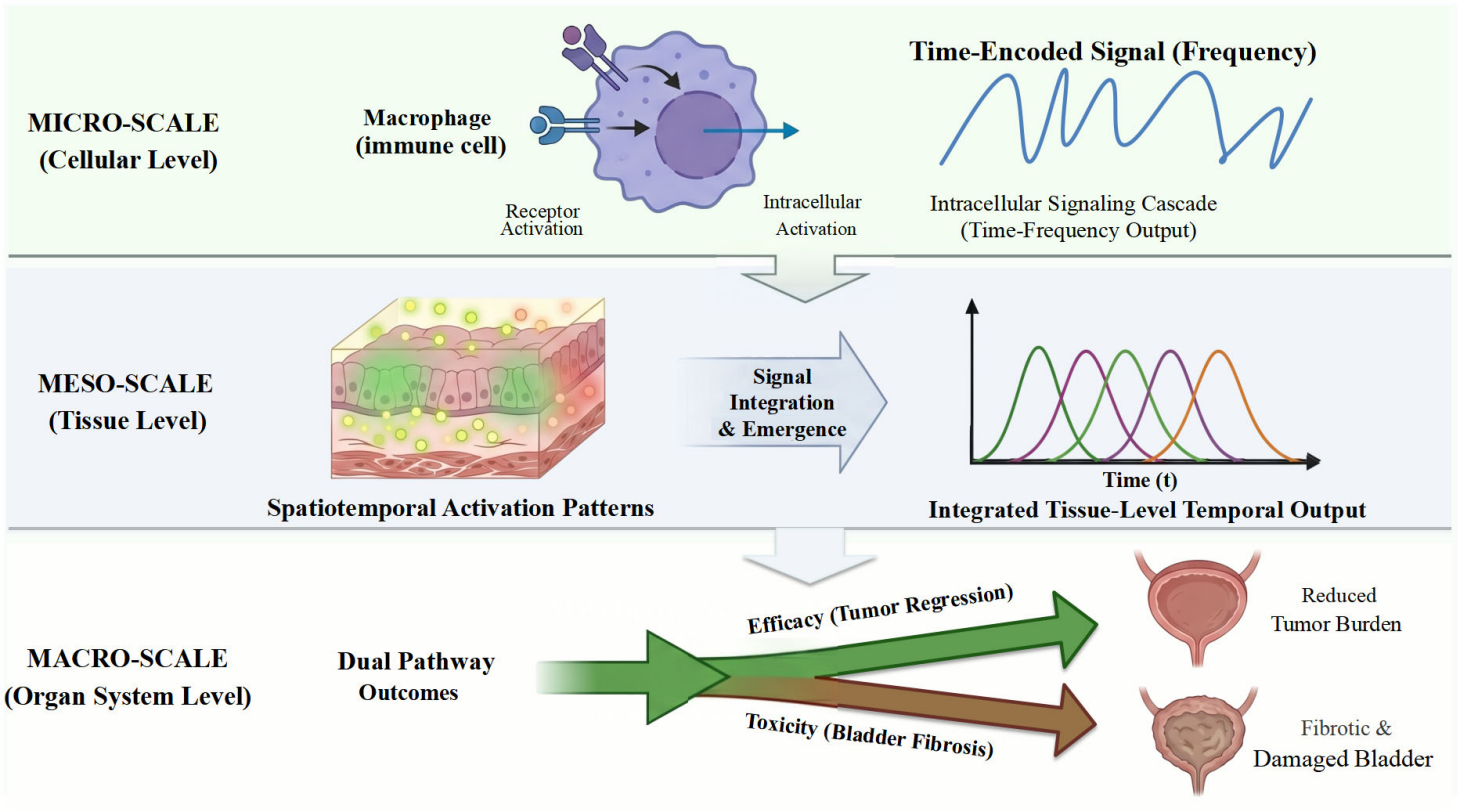
The “micro-meso-macro” framework of innate immune temporal coding. The micro level (left) depicts single-cell signaling dynamics, with a macrophage shown as a representative innate immune cell. The meso level (center) represents the emergent, population-level cytokine waveform accessible in urine. The macro level (right) encompasses long-term oncological and functional outcomes. Additional innate and adaptive cell types participating in the BCG-induced response are discussed in the main text.

These two observational levels sit at the mesoscopic interface of a broader “micro–meso–macro” framework (**Figure 1**). The micro level—single-cell signaling dynamics such as NF-κB oscillations—has firmly established that time is a critical information carrier. The macro level encompasses the long-term oncological and functional outcomes that time-dependent treatment schedules aim to improve. Missing is the link at the mesoscopic level, a population-level readout of local innate immune activity where the temporal dimension is preserved. The urinary cytokine waveforms we define here fill this gap. It is this noninvasive, temporally resolved readout that we hypothesize to carry clinically meaningful information.

Our central hypothesis is that the temporal features of a single-instillation single-cytokine waveform—its time-to-peak, elimination half-life, and decay morphology—carry predictive information independent of its peak amplitude. A sharp peak with rapid decay—a temporally confined signal—might mimic an acute, self-limiting infection and optimally drive Th1 immunity. Conversely, a sustained plateau with slow resolution—a temporally persistent signal—could foster a regulatory or fibrogenic microenvironment. In essence, two cytokine responses of identical peak magnitude may drive fundamentally different adaptive immune outcomes if their temporal shapes differ. Extending this logic across instillations, the longitudinal waveform trajectory may carry additional predictive value beyond any single waveform alone: a trajectory of progressive sensitization could indicate durable immune memory, whereas a trajectory of diminishing responses could signal incipient exhaustion. The two observational levels therefore form a nested, testable substrate for the hypothesis that the temporal dimension of the innate immune response—its waveform—is a clinically meaningful parameter in BCG immunotherapy.”

## 2 Temporal coding of innate immune signals—from single cells to the clinical translation gap

### 2.1 Temporal patterns of innate immunity encode function

Upon recognizing pathogens or damage signals, the innate immune system does not simply “switch on” inflammatory responses; rather, it encodes distinct functional instructions through the kinetic patterns of signal activation. Using real-time single-cell imaging, Nelson et al. first revealed that NF-κB nuclear translocation exhibits autonomous oscillations with a period of approximately 90–120 minutes, and that different oscillation frequencies selectively activate distinct subsets of downstream genes (1). Subsequently, this principle was expanded from a single frequency dimension to a multidimensional “signaling codon” framework: Adelaja et al. decomposed the kinetic trajectory of NF-κB into six quantifiable features—including oscillatory behavior, duration, activation speed, and early/late activity—and demonstrated that different ligands generate distinct codon combinations that drive stimulus-specific transcriptional responses in macrophages (4).

Building on this foundation, two studies provided further support for the principle that “time carries additional predictive value independent of amplitude.” Sen et al., using modeling and single-cell analysis, demonstrated that under a fixed total signal input, merely altering signal duration independently changed both the response repertoire and the number of NF-κB oscillations (5); the “signaling codon” features extracted by machine learning, particularly duration and total activity, were sufficient to drive stimulus-specific gene expression. The same group subsequently discovered that non-oscillatory NF-κB, compared with oscillatory NF-κB, induced greater gains in chromatin accessibility and H3K4me1 enhancer marking, and that these chromatin changes did not fully resolve after stimulus withdrawal but were retained as “epigenetic memory,” enabling cells to mount enhanced immune gene induction upon a second stimulus (6). Together, these two findings reveal that the duration of an immune response not only serves as an immediate functional instruction but also shapes future response capacity.

Notably, the principle of temporal coding is not confined to the NF-κB pathway. Similar temporal decoding logic also operates in adaptive immunity, indirectly pointing to the immunological generality of temporal patterns. T cells likewise exhibit frequency decoding capability: optogenetic experiments have shown that T cells respond most weakly to stimuli with a period of approximately 25 minutes, indicating that their signaling machinery possesses an intrinsic “frequency filter” property (7).

In summary, the principle that innate immune signals encode functional instructions through kinetic features such as oscillation frequency, duration, and decay kinetics has received experimental support at multiple levels—including single-cell signal transduction, epigenetic memory, cross-tolerance regulation, and cross-pathway validation—and has been systematically consolidated into the combinatorial coding and temporal coding framework of innate immunity.

### 2.2 From “time of drug exposure” to “time of host response

The single-cell kinetic principles described above raise a natural clinical question: in human immunotherapy, have we overlooked the kinetic dimension of the endogenous host response? A close examination of current clinical practice reveals that the temporal dimension is not entirely absent. In pharmacology, time-related parameters of drug exposure are a central focus of PK/PD modeling; for instance, dose selection for anti-PD-1 antibodies relies heavily on kinetic analyses of receptor occupancy and plasma drug concentration (8). Clinically, a positive association between the duration of neoadjuvant immunotherapy exposure and the complete response rate has also been preliminarily reported (9).

However, the focus of this “temporal thinking” has remained squarely on the drug side, asking “how long the drug stays in the body.” A qualitatively different dimension—how the host innate immune system, once awakened, mounts its response, how long the response lasts, and when it resolves—has received scant attention. This is precisely the kinetic dimension of the endogenous host response—the temporal shape of how the response is mounted, how long it lasts, and when it resolves—that has remained largely unexamined.

Some clinical trials have begun to capture signals of endogenous host cytokine kinetics: responders to TCR-T therapy exhibited more pronounced IFN-γ and IL-6 elevations on days 3–4 (10); following STING agonist administration, circulating levels of CXCL10, IFN-γ, and IL-6 rose at 2–4 hours and peaked at 6–8 hours (11); and in treatment with the localized IL-2 delivery device AVB-001, most immune markers returned to baseline within approximately 2 weeks (12). However, these observations have thus far remained at the level of single-time-point peak comparisons and have not yet incorporated the full response profile—including elimination half-life and area under the curve—as systematic variables for analysis.

In summary, the current discussion of “time” in tumor immunotherapy presents a “dual presence” pattern: time-related parameters of drug exposure have garnered widespread attention, whereas the temporal shape of the endogenous host immune response—its kinetic dimension—remains at the periphery of the evaluation framework. This article does not deny the importance of drug exposure time; rather, it points out that, beyond drug exposure, the kinetic profile of the endogenous host response may also represent an important dimension of information.

## 3 BCG—a unique system for studying the temporal biology of human innate immunity

### 3.1 From micro-scale signal oscillations to the mesoscopic cytokine profile

Single-cell imaging and modeling analyses have provided molecular-level support for the temporal coding principle of innate immune signaling: kinetic features of NF-κB—such as oscillation frequency, duration, and decay kinetics—drive stimulus-specific gene expression programs (1, 4, 6, 13). Upon entering the bladder lumen, BCG is recognized by pattern recognition receptors (e.g., TLR2/4) on urothelial cells and resident immune cells, activating NF-κB through the MyD88-dependent signaling pathway, and may also involve sensing of mycobacterial DNA via the cGAS-STING pathway (14–17). These signals ultimately converge to initiate transcriptional programs for proinflammatory cytokines, prominently including IL-1β, IL-6, TNF, CCL2, and IFN-γ (18).

However, the focus of this article is not the microscopic details of these molecular events, but rather the holistic, noninvasively observable population-level behavior they give rise to at the tissue/cavity fluid interface. We term this emergent, population-level, noninvasively observable pattern the mesoscopic profile—the cytokine concentration–time curve in urine that reflects the integrated output of local innate immune activity following BCG instillation. This profile does not stand apart from innate immunity; rather, it represents an observational dimension of innate immunity at the *in vivo*, population level that has been previously overlooked.

What connects single-cell signaling events to this mesoscopic population behavior is not a simple linear summation, but a multi-layered biological process. First, the signaling network itself has intrinsic properties that favor spatiotemporal propagation: the combination of rapid positive feedback and delayed negative feedback in the NF-κB network satisfies the conditions for propagating waves in excitable media, predicting that cytokine waves may be a natural mode of signal spread in inflamed tissues (19). Second, chemokine-driven cell recruitment amplifies and sustains the response beyond the timescale of single-cell oscillations: BCG-activated urothelial cells and resident macrophages release chemokines that drive the infiltration of neutrophils and monocytes, which in turn become secondary sources of cytokine secretion (20–22). Third, paracrine communication coordinates this population behavior, as demonstrated by the finding that TNF-α from a small number of high-secreting macrophages can orchestrate IL-6 and IL-10 secretion across the entire population (23). Together, these layers—network excitability, cell recruitment, and paracrine coordination—give rise to an emergent, population-level waveform at the tissue level. The urinary cytokine curve is therefore not a direct readout of intracellular NF-κB oscillations, but the integrated output of a multi-step tissue process, carrying information that is not present in any single-cell trace alone.

Once formed, this mesoscopic profile constitutes the dynamic microenvironment encountered by subsequently infiltrating immune cells, and shapes the efficiency of dendritic cell maturation, antigen cross-presentation, and Th1 polarization—thereby serving as a functional interface through which innate immune kinetics output to adaptive immunity (24, 25). Moreover, the waveform itself is not merely a passive record: as shown by Son et al., the spatial range and oscillation number of NF-κB activation are independently determined by the secretion duration of the source cell (26, 27), implying that decay rate and other temporal features are active information carriers sensed by newly arriving immune cells. It is this mesoscopic profile—operationalized as the single-instillation single-cytokine waveform—that we propose as the core, noninvasively accessible readout for testing whether innate immune temporal coding principles operate in the human body.

### 3.2 Urinary cytokines and adaptive immunity: the temporal dimension of BCG-induced immune responses

The causal role of cytokine temporal waveform in shaping adaptive antitumor immunity has been experimentally demonstrated. Zemek et al., using a bilateral tumor model, showed that two IFN-β kinetic patterns—”on/fast-off” versus “persistent-on”—produced divergent ICB responses. Crucially, when they exogenously mimicked these patterns using identical cytokine, dose, and route during the first three days, varying only signal duration thereafter, the pulsed (fast-off) regimen enhanced CD8^+^ T cell infiltration and ICB efficacy while the chronic regimen did not (28). This establishes that the temporal waveform of an innate cytokine can alone determine whether an effective adaptive T cell response is mounted.

This principle raises a focused question: could analogous waveform–function coupling operate in intravesical BCG therapy, where the innate-to-adaptive cascade is well characterized?

Following intravesical instillation, BCG is internalized by urothelial cells, resident macrophages, and dendritic cells (DCs), and can also directly infect bladder cancer cells, inducing them to present antigens and secrete cytokines (29). This initiates a coordinated innate-to-adaptive cascade. BCG drives M2-to-M1 macrophage repolarization, shifting the local milieu from an IL-10-dominated, immunosuppressive state to an IL-12-dominated environment that favors Th1 immunity (30, 31). Activated DCs upregulate CD80, CD86, CD83, and MHC class II molecules and migrate to draining lymph nodes to prime naïve CD4^+^ T cells (32, 33). Within this cascade, specific cytokines serve as direct instructors of adaptive response quality: IL-12 is the master Th1-polarizing cytokine (30, 32); IL-18 synergizes with IL-12 to amplify IFN-γ production by T cells and NK cells (34, 35); IL-1β and TNF-α promote DC maturation and antigen-presenting capacity (33). At the effector stage, IFN-γ suppresses Th2 responses and activates macrophage tumoricidal activity, while the chemokine CXCL10 (IP-10) establishes a gradient that recruits CXCR3^+^CD8^+^ T cells, Th1 cells, and NK cells into the bladder mucosa (36). Concurrently, BCG-induced trained immunity—epigenetic and metabolic reprogramming of innate cells that enhances secondary cytokine responsiveness—provides a mechanism for sustained immune memory lasting 6–12 weeks (37). Thus, the cellular and molecular machinery required to relay an innate immune signal and orchestrate an adaptive antitumor response is unequivocally present in the bladder wall during BCG therapy.

Importantly, the cytokine response triggered by BCG instillation is not temporally uniform. A comprehensive review of BCG immunotherapy documents a dynamic, phasic cytokine cascade following each instillation (38). Within hours, pro-inflammatory cytokines such as IL-1, IL-6, IL-8, and TNF-α surge, predominantly from macrophages and neutrophils. Within 24 hours, IL-12 and IFN-γ become detectable alongside sustained IL-1 and IL-6, indicating early T cell and NK cell participation. Across repeated instillations, distinct cytokines display different peak kinetics: IL-1β and CCL2 culminate around the third instillation, whereas IFN-γ reaches its maximum at the fourth, before a gradual counter-regulatory tone (IL-1Ra, IL-10) emerges during the maintenance phase. These observations confirm that BCG-induced urinary cytokines are not simply “on” or “off” but exhibit time-dependent rise-and-fall trajectories. This temporal heterogeneity raises a testable question: do distinct single-instillation single-cytokine waveform—a sharp peak with rapid decay versus a sustained plateau—differentially instruct the quality of downstream adaptive immunity and, ultimately, clinical outcome?

### 3.3 Clinical hints of cytokine waveform following BCG instillation

The hypothesis that waveform features may be associated with clinical outcomes has not been prospectively tested. However, a body of BCG clinical studies—though designed primarily to measure cytokine magnitude—has inadvertently captured fragments of temporal information. **Table 1** summarizes these studies. Critically, none was designed to test waveform features as prespecified endpoints; their sampling protocols were optimized for single-time-point or sparse comparisons, not for characterizing full rise-and-fall kinetics. Nevertheless, the temporal patterns that emerge from these data are directionally consistent with the waveform hypothesis and merit critical appraisal.

**Table 1:** Prospective BCG instillation studies providing temporal cytokine information.

Cytokines measured	Sampling time points	Key temporal finding	Ref
IL-1α, IL-1β, IL-2, IL-6, TNF-α, IFN-γ	Pre, 2, 4, 6, 8, 24 h (weekly ×6)	Systematic differences in time-to-peak and decay rate across cytokines	(39)
IL-6, IL-2	Pre, 1, 2, 3, 6, 12, 24 h (weekly ×6)	Three IL-6 response patterns identified (early/delayed/non-response); IL-6 levels correlated with IL-2 induction and neutrophil count	(40)
IL-8, IL-18	0–6 h (IL-8); 0–12 h (IL-18) after 1st instillation	IL-8 >4,000 ng/6h associated with >36-month disease-free survival; IL-18 significantly elevated in recurrence-free patients	(34)
IL-1β, IL-2, IL-6, TNF-α, IFN-γ, M-CSF	Urine: Pre, 6–8 h (weekly ×6)	Week of peak level differed by cytokine (IL-1β week 3, IFN-γ week 4, TNF-α weeks 5-6)	(41)
GM-CSF, TNF-α, G-CSF, IL-1β, IL-8, IFN-γ, IL-12	1st & 6th instillation: Pre, 4, 8, 24 h	Greater increase in TNF-α Cmax at 6th vs. 1st instillation (sensitization-type longitudinal trajectory); increment associated with recurrence-free outcome	(42)
IL-2	2–4 h & 4–6 h (max), weekly ×6 (repeated courses)	Short interval (≤12 months) shifted IL-2 peak to earlier weeks and reduced week-6 levels; interval positively correlated with peak week (r = 0.81)	(43)
IL-2, IL-10 (urine); TNF-α, CTLA-4, transcription factors (serum)	Baseline, 4 h post-6th	TNF-α↑ predicted ICR; IL-10↑, CTLA-4↑, T-bet+↓, FoxP3+↑, GATA3+/T-bet+↑ predicted recurrence and progression	(44)
12 cytokines (IL-2, IL-6, IL-8, IL-10, IL-12, IL-18, TNF-α, etc.)	Baseline; wk6 (pre & 4h post); maintenance cycle 3 (pre & post)	Single-cytokine changes associated with recurrence, but multi-cytokine combination achieved best predictive performance	(45)

All studies used urine as the primary sample type. Studies 4, 7 also included serum/PBMC as specified. Abbreviations: DFS, disease-free survival; ICR, initial complete response.

Three themes emerge from these studies. First, inter-individual and inter-cytokine differences in timing have been repeatedly documented. As early as 1992, De Boer et al. showed that different cytokines exhibit distinct time-to-peak and decay profiles, and Schamhart et al. subsequently identified three patient subgroups based on their temporal response patterns—early, delayed, and non-responsive (39, 40). Thalmann et al. later reported that IL-8 elevation within 6 hours and IL-18 elevation within 12 hours were associated with favorable prognosis, and Taniguchi et al (34, 41). found that the week of peak cytokine levels differed by cytokine, suggesting that timing, not merely magnitude, carries prognostic information.

Second, longitudinal trajectory across instillations has been observed. Shintani et al. demonstrated that TNF-α levels at 4 hours post-instillation increased from the first to the sixth week, and that this increment was associated with recurrence-free outcomes—a pattern consistent with what we now term a “sensitization-type” longitudinal waveform trajectory (42). De Reijke et al., tracking patients across repeated treatment courses, found that shorter inter-course intervals shifted the week of IL-2 peak earlier and reduced late-course levels, suggesting an “exhaustion-type” trajectory (43). Elsawy et al. added that longitudinal patterns of urinary IL-10, rather than single-time-point levels, were independently associated with recurrence and progression (44).

Third, multi-cytokine combinations may capture waveform complexity beyond single parameters. Kamat et al.’s CyPRIT nomogram, which integrates changes in multiple cytokines, outperformed individual cytokine levels in predicting recurrence—hinting that a composite waveform signature may be more informative than any single amplitude or timing measure (45).

These observations must be interpreted with caution. None of these studies was designed with a prespecified waveform hypothesis; their sampling schedules were sparse (typically 1–4 time points per instillation), making it impossible to reliably calculate parameters such as half-life or to distinguish a sharp peak from a broad plateau. Cohorts were small, BCG strains and schedules heterogeneous, and assay methods variable. Inconsistent correction for urine dilution and the absence of baseline normalization further limit comparability. Consequently, the patterns summarized here serve only as directional support for the waveform hypothesis—they are not confirmatory evidence. Direct prospective testing, with high-temporal-resolution sampling and prespecified waveform parameters as endpoints, is required to determine whether the temporal dimension of the innate immune response carries independent clinical value.

## 4 The bladder—a window for decoding immune response kinetics

### 4.1 The bladder as a potential model for decoding innate immune kinetics

By virtue of its unique physiological and anatomical advantages, the bladder may serve as a potential model for decoding innate immune kinetics.

First, the input signal is known and standardized. The standard BCG regimen is clearly specified in EAU and AUA guidelines: a 6-week induction phase with once-weekly instillations, followed by maintenance once every 3 months for 1–3 years (46, 47). This renders the “moment of stimulation” a pre-known, uniform condition. In contrast, the timing and intensity of endogenous immune-activating events in solid tumors—such as antigen release upon tumor necrosis—are unpredictable, making them difficult to use as “controlled stimuli” for studying response kinetics. Although BCG preparations exhibit biological potency variations across strains and batches (48), the strain, dose, and frequency used in a given clinical study are well documented, ensuring their comparability as input variables. It is worth noting that, owing to toxicity and adherence issues in clinical practice, actual instillation regimens often deviate from guideline recommendations—studies show that only 41.5% of patients complete the full induction plus maintenance protocol (49)—yet this variability is not a mere confounder. It can be recorded and incorporated as an input variable into kinetic models, thereby providing natural-experiment data from the real world for investigating “the impact of different time intervals on the immune response.”

Second, the output signal can be sampled noninvasively via urine with high temporal resolution. Dynamic monitoring of the tumor microenvironment in solid tumors has long faced a fundamental dilemma: blood-based biomarkers reflect systemic immune status but are difficult to localize to the tumor site; tissue biopsies provide local information but cannot be repeated at high frequency; and imaging has a temporal resolution on the order of weeks to months, far too coarse to capture the hour-to-day dynamics of innate immune responses. Pleural and peritoneal effusions can be obtained via puncture, but sampling frequency is constrained by the invasiveness of the procedure, making it difficult to collect samples routinely before and after each immune stimulus. The bladder overcomes this impasse: in non-muscle-invasive bladder cancer, the tumor resides in the urothelial layer, and urine is in direct contact with the tumor surface. By collecting voided urine samples at pre-specified time points—for example, at 2, 4, 6, 8, and 24 hours post-instillation—one can obtain a dynamic curve of the BCG-triggered immune response.

Finally, clinical endpoints can be evaluated in a stratified manner within the same model. The clinical outcomes of BCG intravesical instillation can be assessed along two distinct dimensions: oncological outcomes (recurrence, progression, cystectomy, tumor-specific survival) and bladder function outcomes (decreased bladder capacity, storage-phase symptoms such as urinary frequency and urgency, and bladder contracture). This dual-endpoint design enables investigators to examine separately the associations between kinetic parameters of the immune response and different outcomes—for example, whether peak intensity correlates with antitumor efficacy, and whether the decay rate correlates with post-inflammatory fibrosis. It is precisely within this closed loop—”controlled stimulus—noninvasive high-frequency sampling—dual endpoints”—that the intravesical bladder model possesses unique advantages, establishing it as a potential model for testing the waveform hypothesis in the human body.

### 4.2 From hypothesis to operation: a prospective validation framework

Translating the waveform hypothesis into a testable proposition requires a prospective cohort explicitly designed to capture temporal dynamics.

Patients with high-risk NMIBC scheduled for standard BCG induction and maintenance would be enrolled. During the 6-week induction phase—the most immunologically dynamic period encompassing innate priming, peak cytokine production, and establishment of trained immunity—all six weekly instillations receive high-resolution sampling. Within each instillation, we propose six target sampling time points: pre-instillation (0 h, individualized baseline) and post-instillation at 2, 4, 6, 8, and 24 h. These time points are informed by protocols from prior studies that documented detectable cytokine dynamics (**Table 1**) and by systematic summaries of BCG-induced urinary cytokine kinetics (38): 2 h captures pioneer cytokine emergence; 4–6 h brackets the peak aggregation window for most acute-phase cytokines; 8 h discriminates rapid decay from a sustained plateau; and 24 h assesses baseline reset.

**Figure 2:**
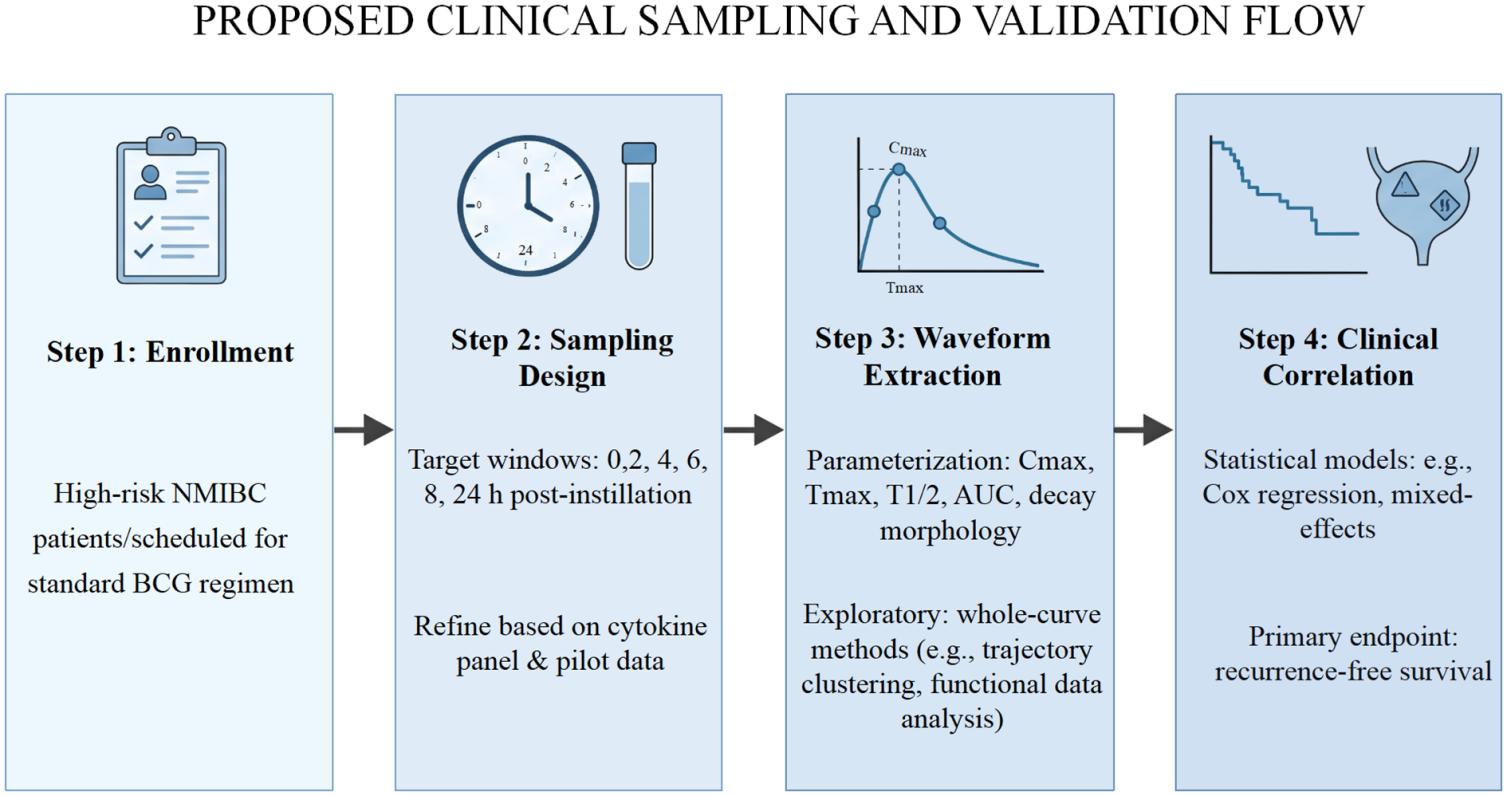
Summarizes this validation pathway from enrollment through clinical correlation.

In practice, rigid adherence to clock times is often constrained by variable bladder capacity and BCG-induced irritative symptoms. We therefore recommend target windows (e.g., 1.5–2.5 h for the 2 h time point) rather than fixed time points, with the exact post-instillation voiding time recorded as a continuous variable. Specimens obtained outside these windows remain analyzable, as e.g., mixed-effects models can accommodate irregularly spaced longitudinal data. A minimum dwell time (e.g., <1 h) may be used as an exclusion criterion in sensitivity analyses. This approach trades a modest relaxation of temporal standardization—which can be recovered analytically through exact time recording—for a meaningful gain in clinical feasibility. The overall validation pathway is summarized in **Figure 2**.

The choice of priority cytokines should be guided by the specific hypothesis being tested and the temporal position of each factor in the innate-to-adaptive cascade (Section 3.2). Acute-phase pioneer cytokines (e.g., IL-8, TNF-α) may be prioritized for hypotheses concerning innate sensing speed; factors at the Th1-polarization interface (e.g., IL-12, IFN-γ) for hypotheses concerning adaptive effector quality; and resolution-phase regulators (e.g., IL-10, IL-1Ra) for hypotheses concerning the immune reset window.

Urinary cytokine concentrations should be normalized to urinary creatinine measured in the same sample to correct for dilution. The pre-instillation (0 h) value of each cycle serves as the cycle-specific baseline: Cmax and AUC are computed as net increments above this baseline; Tmax and T1/2 are calculated directly from the creatinine-normalized concentration-time curve. Gross hematuria, common after BCG instillation, may interfere with certain immunoassays and should be recorded for sensitivity analyses.

The above design parameters—sampling time points, cytokine panel composition, and normalization strategy—represent a reference framework derived from currently available evidence. In practice, they can and should be refined according to the specific experimental objectives, statistical modeling requirements, and results of initial feasibility testing.

### 4.3 Waveform quantification and statistical analysis

**Figure 3:**
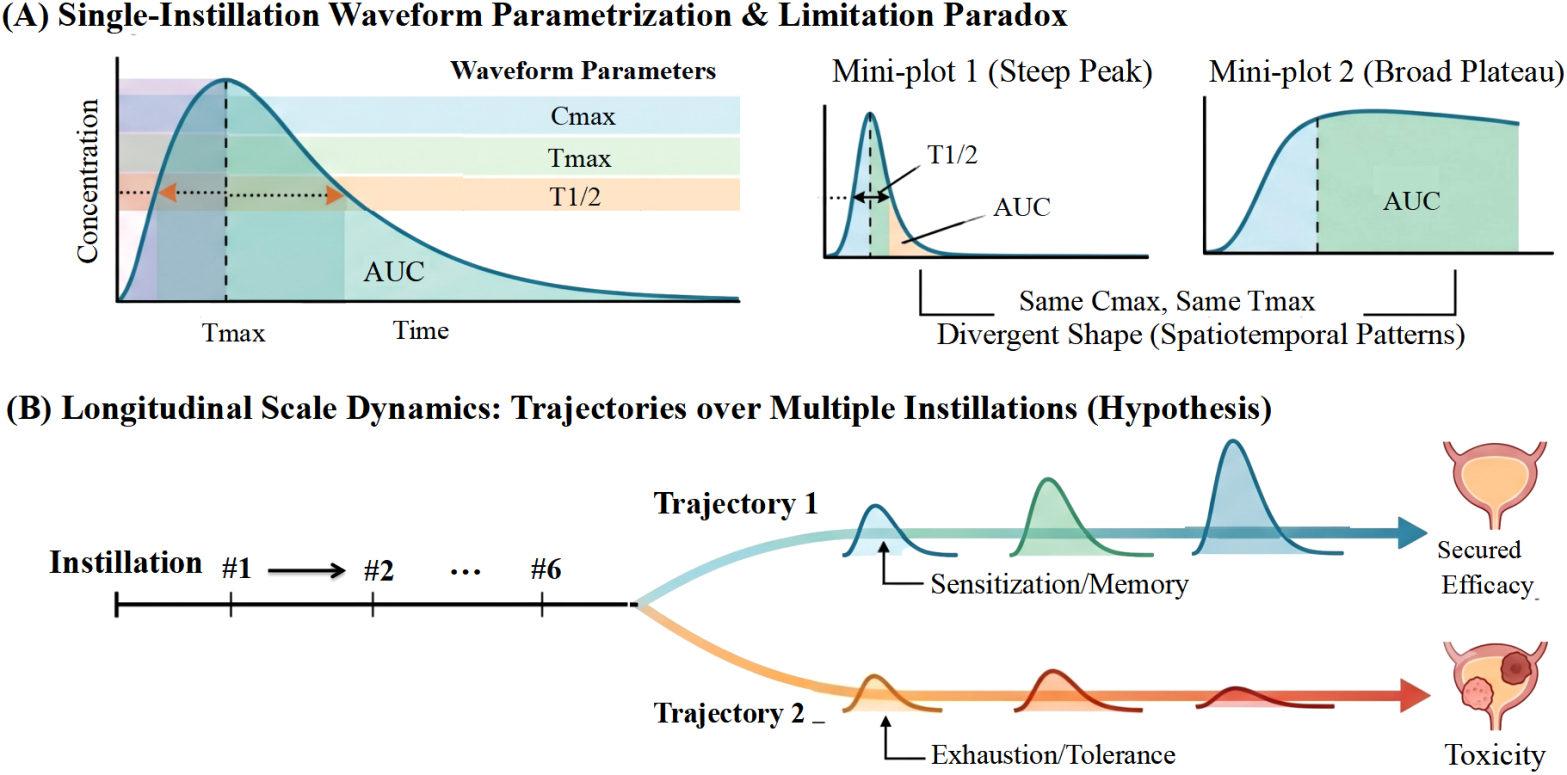
Conceptual framework for waveform parameterization and longitudinal trajectory evaluation. **(A)** Single-instillation kinetic parameters and shape divergence; **(B)** Longitudinal trajectories contrasting sensitization versus exhaustion.

The waveform hypothesis requires analytical approaches that treat the cytokine time curve as a whole. The most direct strategy is parameterization: distilling each single-instillation single-cytokine curve into Cmax (peak concentration), Tmax (time to peak), T1/2 (elimination half-life), and AUC (total exposure), using the pre-instillation value as the individualized baseline. The core testable proposition is whether Tmax or T1/2 independently predicts clinical outcomes after adjusting for Cmax. Two caveats accompany this approach: waveforms of fundamentally different shapes can yield identical parameter values, and AUC is mathematically coupled with Cmax, requiring variance inflation factor assessment in multivariable models. The conceptual framework for both single-instillation waveform parameterization and the longitudinal trajectories across repeated instillations is illustrated in **Figure 3**.

Complementary whole-curve methods can capture waveform information beyond individual parameters. Trajectory clustering can group patients into distinct waveform archetypes (e.g., “spike-and-resolve” vs. “sustained-plateau” vs. “delayed-rise”) without relying on pre-specified parameters (50, 51). Functional data analysis, by fitting continuous curves to discrete measurements, can extract features such as rate of change and time to baseline return (52). These approaches are exploratory and serve as hypothesis-generating complements to parameter-based testing.

Linking waveform features to clinical endpoints requires models appropriate to the study design. For single-instillation waveform shape, Cox proportional hazards models with waveform parameters as primary predictors, adjusted for Cmax and established clinical covariates, provide a straightforward framework. Mixed-effects models provide the foundation for characterizing how cytokine features evolve across instillations, accommodating fixed effects (e.g., instillation number, time since instillation) and individual-level random effects (53). To directly test whether this longitudinal evolution predicts clinical outcomes, joint longitudinal-survival models are particularly well-suited: the longitudinal submodel describes the cytokine trajectory, and the survival submodel relates it to recurrence or progression, treating the waveform trajectory as a time-dependent exposure (54). Regardless of the analytical approach, the multiplicity of cytokines, time points, and waveform features inflates the risk of false discovery; prespecification of the primary hypothesis and appropriate correction (e.g., Benjamini-Hochberg) are essential (55).

**Table 2** summarizes provisional, mechanistically informed hypotheses linking specific waveform features to putative biological processes and testable clinical endpoints. These associations are illustrative; in practice, each hypothesized link must be specified per cytokine, as a given waveform feature may carry different biological meanings for different molecules.

**Table 2:** Provisional hypotheses linking waveform features to biological processes and clinical endpoints.

Waveform feature	Putative biological process	Hypothesized clinical association
Single-instillation parameters		
Cmax	Magnitude of local innate activation	Higher Cmax: lower recurrence risk, but greater local toxicity
Tmax	Speed of cell recruitment and signal amplification	Shorter Tmax: more efficient innate readiness; longer recurrence-free survival
T1/2	Rate of inflammatory resolution	Prolonged T1/2: bladder function deterioration (sustained inflammation → fibrosis)
AUC	Cumulative inflammatory pressure	High AUC: reflects excessive inflammatory exposure; may associate with increased bladder toxicity. Relationship with recurrence may be non-linear.
Decay morphology		
Exponential decay	Active resolution	Favorable for both tumor control and bladder preservation
Biphasic decay	Ongoing immune activity	May reflect sustained paracrine amplification
Longitudinal trajectory		
Sensitization (Cmax↑ across instillations)	Trained immunity; epigenetic reprogramming	Durable response to maintenance therapy
Exhaustion (Cmax↓ across instillations)	Innate tolerance; counter-regulatory dominance	Limited benefit from continued maintenance

All associations are provisional hypotheses for prospective testing.

The waveform hypothesis makes a falsifiable prediction: temporal features of the cytokine response should carry clinical information independent of response magnitude. The strongest support would come from convergence across analytical strategies—for instance, if trajectory clustering identifies waveform archetypes that segregate patients by recurrence status and are also distinguishable by their parameter profiles. Conversely, the absence of any detectable temporal signal across multiple analytical frameworks would substantially weaken the hypothesis.

## 5 Limitations and challenges

The waveform hypothesis proposed in this article rests on premises that await empirical testing. Below we address two categories of limitation: those arising from the evidence base and the urinary readout, and those defining the boundaries within which the hypothesis can meaningfully apply.

### 5.1 Uncertainty in the evidence base and the urinary readout

The clinical studies summarized in Section 3.3 and **Table 1** provide directional support for the waveform hypothesis, but they were not designed to test it. All have important limitations: small cohorts, heterogeneous BCG strains, doses, and schedules, variable assay platforms, and—most critically—sampling protocols that were optimized for single-time-point or sparse comparisons, not for characterizing complete rise-and-fall kinetics. None prespecified waveform parameters as endpoints. The temporal patterns they reveal are therefore fragmentary; they suggest that timing may matter but cannot confirm or refute the specific hypothesis advanced here. These observations are best viewed as motivation for prospective studies, not as evidence that the waveform framework is clinically valid.

Beyond these limitations in the clinical evidence, there are intrinsic uncertainties in the urinary readout itself. Urinary cytokine concentrations are influenced not only by local bladder secretion but also by systemic filtration, urine dilution, voiding frequency, and bladder capacity (56, 57). Although normalization to urinary creatinine or osmolality can mitigate some of this noise, both methods assume stable renal function and a constant relationship between the dilution marker and cytokine concentration—assumptions that may not hold in the peri-instillation period, particularly if BCG-induced cystitis alters urine handling. Gross hematuria, a common finding after BCG instillation, may further interfere with certain immunoassays (58). A more fundamental issue is that voided urine is a discontinuous sample of a continuous process: the single−instillation single−cytokine waveform measured in urine is inherently a temporally smoothed and delayed representation of the underlying tissue dynamics, and rapid or biphasic events at the tissue level may be unresolvable. Before proceeding to large-scale human studies, animal models in which tissue and urine are simultaneously sampled would help establish the fidelity with which the urinary waveform reflects local immune events. These uncertainties do not render the urinary waveform useless as a surrogate readout; they do, however, require that the urinary waveform be interpreted as an approximate readout of the local tissue response rather than as a precise mirror of tissue biology.

### 5.2 Theoretical gaps and tumor-intrinsic boundaries

Beyond the methodological considerations above, the waveform hypothesis has a clearly defined explanatory boundary: it concerns the delivery of immune pressure, not the tumor’s sensitivity to it.

The framework proposed in this article addresses how the temporal shape of the innate immune response shapes the initiation, polarization, and functional quality of adaptive immunity (Section 3.2). If validated, waveform optimization could maximize the intensity and temporal precision of immune pressure—ensuring that the right type of effector response is generated at the right time. However, immune pressure is only one half of the equation. The antitumor effect ultimately requires that the generated effector cells encounter a tumor that is capable of being recognized and eliminated.

Tumor-intrinsic resistance mechanisms constitute an effector checkpoint downstream of immune activation, and they operate independently of waveform quality. Well-characterized examples include loss-of-function mutations or epigenetic silencing of HLA class I molecules and β2-microglobulin, which render tumor cells invisible to CD8^+^ T cells regardless of how efficiently those T cells have been primed. Similarly, upregulation of PD-L1 on tumor cells can directly inhibit effector T cell function at the tumor bed, effectively neutralizing even a robust Th1-polarized infiltrate (59, 60). Other mechanisms, such as defects in IFN-γ receptor signaling or overexpression of anti-apoptotic proteins, further raise the threshold for immune-mediated elimination (61). In all these scenarios, the waveform may be “correct”—a brisk, appropriately resolved innate response that drives potent Th1 immunity—yet tumor clearance fails because the effector checkpoint is blocked.

This distinction carries an important implication: waveform parameters and tumor-intrinsic resistance markers provide non-redundant information. The waveform describes the strength and temporal pattern of the immune pressure applied; tumor-intrinsic features describe whether the target is susceptible to that pressure. These two dimensions can, in principle, be jointly assessed. Tumor stage, grade, and CIS status capture disease burden and biological aggressiveness, while prior BCG exposure history and baseline immune cell infiltration reflect the immunological starting state of the tumor microenvironment. Superimposed on these are the molecular resistance mechanisms discussed above—HLA class I loss, PD-L1 upregulation, and IFN-γ pathway defects—which define the effector checkpoint more directly (62). A clinically useful predictive model would likely need to integrate both dimensions, with waveform classification (e.g., “optimal” vs. “suboptimal” archetypes) contributing the temporal immune-pressure axis and established tumor-intrinsic biomarkers contributing the sensitivity axis. Whether this integration improves risk stratification beyond either dimension alone is an empirical question for future studies and lies beyond the scope of the current hypothesis.

## 6 Discussion

This article brings together three categories of evidence that differ substantially in their level of certainty. What is firmly established is the principle that temporal patterns—oscillation frequency, duration, and decay kinetics—encode functional instructions in innate immune signaling. This has been mechanistically dissected at the single-cell level, and a causal link between cytokine temporal waveform and adaptive immune outcome has been experimentally demonstrated by Zemek et al. What existing clinical observations suggest, but do not confirm, is that BCG-induced urinary cytokines exhibit temporally structured waveform shapes with inter-individual heterogeneity, and that this heterogeneity may be associated with clinical outcomes. The studies providing these hints were designed to measure cytokine magnitude at selected time points, not to characterize complete rise-and-fall kinetics or to test waveform shape as a prespecified predictor; their findings therefore serve as motivation for hypothesis-driven prospective testing rather than as confirmatory evidence. What remains an untested hypothesis is the proposition at the core of this article: that the shape of a single-instillation single-cytokine waveform—the integrated pattern of rise, peak, and decay—carries clinically meaningful information, and that distinct waveform archetypes may differentially shape adaptive immunity and point toward divergent clinical outcomes. This three-tiered structure—established mechanism, indirect clinical hints, and untested hypothesis—defines the evidentiary gap that prospective studies must now address.

The framework presented here deliberately takes the single-instillation single-cytokine waveform as its fundamental unit of analysis. This is a methodological choice, not a conceptual limitation. Before a multi-cytokine kinetic profile or a patient-specific composite signature can be meaningfully constructed, it is necessary to first establish that individual cytokine waveforms carry clinically relevant temporal information. Once waveform features have been validated for a core panel of cytokines—for instance, IL-8 for innate sensing speed, IL-12 and IFN-γ for Th1 polarization quality, and IL-10 for resolution kinetics—two higher-order constructs become accessible. A multi-cytokine kinetic profile could integrate temporally aligned waveforms from multiple cytokines into a coordinated signature of immune response quality. Further along this path, a patient-specific composite immune-response signature could incorporate waveform parameters with baseline clinical variables and tumor-intrinsic biomarkers to enable individualized risk stratification. We therefore advocate a sequential validation strategy: first establish the clinical relevance of single-cytokine waveform features, then integrate them into multi-cytokine profiles, and finally construct patient-level composite models.

If the waveform hypothesis is validated in prospective studies, it would open several lines of investigation. The most immediate concerns patient stratification. Currently, all patients with high-risk NMIBC are recommended to complete a three-year maintenance course, yet only a subset derives durable benefit. If distinct longitudinal waveform trajectories can be identified during the six-week induction phase—for example, a sensitization-type trajectory characterized by progressively increasing Cmax and shortening Tmax, versus an exhaustion-type trajectory characterized by blunted or decelerating responses—these trajectories may distinguish patients who are establishing durable innate immune memory from those whose immune system is becoming refractory to repeated stimulation.

**Figure 4:**
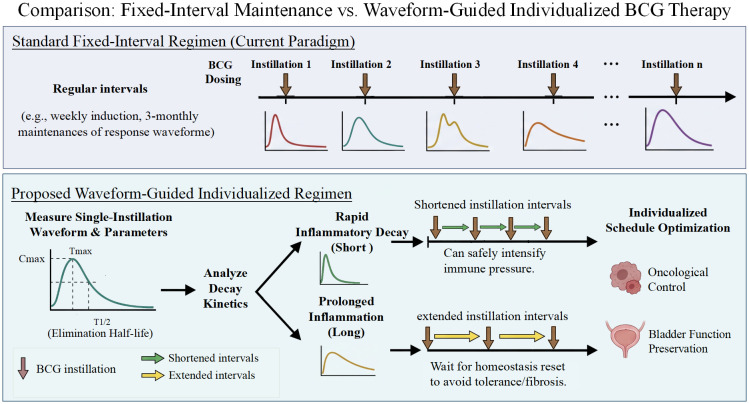
Schematic comparison of fixed-interval maintenance therapy versus waveform-informed individualized instillation scheduling.

A second line of investigation concerns the potential for individualized instillation intervals. The current fixed schedule—weekly induction, followed by maintenance every three months—was empirically derived and does not account for inter-individual differences in the tempo of immune reset. If the decay kinetics of key cytokines can be reliably measured, two scenarios become testable. For a patient whose urinary cytokines return to baseline rapidly after each instillation (short T1/2), a shorter inter-instillation interval might intensify immune pressure without superimposing stimuli on unresolved inflammation. Conversely, for a patient with prolonged cytokine elevation (long T1/2), a longer interval would allow full resolution before the next instillation, potentially avoiding tolerance induction. Such waveform-informed scheduling would differ fundamentally from the current fixed-interval paradigm, as illustrated conceptually in **Figure 4**. It could also address the pragmatic challenge of BCG shortage: if some patients can maintain therapeutic efficacy with extended intervals, the limited BCG supply could be allocated more efficiently. More speculatively, if a detrimental waveform archetype were identified, the question arises whether it could be therapeutically reshaped. Zemek et al. have provided proof-of-principle that exogenously manipulating cytokine kinetics can restore antitumor immunity, raising the possibility of analogous strategies in BCG therapy should a causal waveform-outcome relationship be established.

In summary, this article proposes a testable framework that places the temporal dimension of the innate immune response at the center of BCG pharmacodynamic evaluation. By making the waveform a prespecified object of study, the framework offers the possibility of transforming the assessment of intravesical immunotherapy from an amplitude-only paradigm to an amplitude-plus-waveform paradigm.

## Data Availability

The original contributions presented in the study are included in the article/supplementary material. Further inquiries can be directed to the corresponding author.
